# Access to emergency medical services and associated barriers among war-affected patients evacuated from Gaza: a cross-sectional study

**DOI:** 10.1186/s13690-025-01734-w

**Published:** 2025-10-01

**Authors:** Melih Çamcı, Sueda Zaman, Nourhan Hassanien, Muhammed Fatih Baran

**Affiliations:** 1 Department of Emergency Medicine, Ankara Bilkent City Hospital, Ankara Yıldırım Beyazıt University, Ankara, Türkiye; 2 Department of Emergency Medicine, Mamak State Hospital, Ankara, Türkiye; 3 Turkish Red Crescent, Ankara, Türkiye; 4 Department of Family Medicine, Konya City Hospital, University of Health Sciences, Konya, Türkiye

**Keywords:** Emergency medical services, Health services accessibility, Warfare and armed conflicts, War-related injuries, Gaza strip, Cross-sectional studies

## Abstract

**Background:**

This study aims to evaluate civilians’ experiences in accessing emergency medical services (EMS) within the ongoing conflict in Gaza and to identify the structural, systemic, and human rights-related barriers encountered throughout this process. The literature provides extremely limited quantitative field data focusing on the emergency healthcare experiences of war-affected individuals medically evacuated from regions under siege to other countries. In this respect, the study seeks to contribute to the understanding of possible impairments to the right to health, based on patient experinces during an ongoing conflict situation, while also analyzing measurable access-related factors such as comorbidities, evacuation timelines, and infrastructure adequacy.

**Methods:**

This cross-sectional study included 81 patients medically evacuated from Gaza to Türkiye for treatment due to the escalation of violence following the events of October 7, 2023. Ethical approval was pre-obtained, and written informed consents were secured from all participants. Structured questionnaires were administered, collecting sociodemographic data, medical history, barriers to access, and responses concerning human rights. Descriptive analyses, Chi-square tests, and Spearman correlation analyses were employed for statistical evaluation.

**Results:**

The study included 81 participants, predominantly female (60.5%), with a median age of 26 years. The most frequent conditions were malignancies (35.4%) and extremity trauma (34.1%). A total of 74% reported that healthcare facilities were damaged during the conflict, and 87.6% cited severe equipment shortages in emergency departments. Ambulance services never arrived for 34.5%, and 33.3% could not reach hospital EDs. Patients with comorbidities experienced significantly longer wait times for both ambulance arrival (*p* = 0.013) and ED access (*p* = 0.001). Those waiting over 30 min for ambulances had longer ED treatment durations (*p* = 0.036), whereas delays in reaching the ED were paradoxically associated with shorter in-department stays (*p* = 0.026).

**Conclusion:**

This study identifies measurable weaknesses in Gaza’s emergency healthcare system, including ambulance inaccessibility, delays in reaching emergency departments, and widespread shortages of equipment and personnel. These findings, derived from patient-reported data, reflect systemic barriers to effective emergency care. By identifying specific gaps in infrastructure, care delivery, and coordination, the study offers evidence-based insights to inform targeted humanitarian interventions in conflict settings.

**Supplementary Information:**

The online version contains supplementary material available at 10.1186/s13690-025-01734-w.

**Table:** 

**Text box 1. Contributions to the literature**
• This study offers rare, field-based quantitative data on emergency medical service (EMS) access among war-affected civilians, based on patient-reported experiences during medical evacuation.
• It documents previously unreported indicators, such as ambulance inaccessibility (34.5%), prolonged emergency department delays, and infrastructure inadequacy (e.g., 87.6% citing equipment shortages).
• The study reveals how EMS are obstructed through denial of ambulance access, destruction of health facilities, and widespread shortages of personnel and supplies.
• Findings highlight the urgent need for mobile field hospitals, international monitoring mechanisms, and direct provision of ambulance, equipment, and medication support to ensure protected and equitable EMS access in conflict zones.

## Introduction

The ongoing war in Gaza Strip underscores the critical importance of securing civilian health and safety in conflict zones and ensuring uninterrupted access to emergency medical services (EMS) [1, 2]. In war-affected areas under continuous bombardment, the systematic targeting of healthcare infrastructure severely obstructs civilians’ access to basic medical services [3, 4]. The long-term effects of displacement and occupation have profoundly impaired the Palestinian healthcare system—particularly in areas under siege—where medical centers are directly attacked, ambulances are prevented from reaching the injured, and access to medical supplies is heavily restricted [1, 2]. This multifaceted collapse of the healthcare system inevitably disrupts treatment for patients with chronic diseases, hampers infectious diseases management, and irreversibly affects urgent care for trauma patients. In their study, Beiraghdar et al. (2023) observed a substantial increase in infectious diseases since the onset of the conflict, reporting over 71,000 cases of scabies and lice, 1,005 cases of chickenpox, and 54,866 upper respiratory infections [2].

Under international law, EMS must remain neutral and accessible, even during armed conflicts. However, in Gaza, these services are systematically obstructed: emergency medical personnel are exposed to violence, and both ambulances and healthcare facilities are targeted, effectively restricting access to life-saving care [1, 5]. While most pre-hospital emergency systems worldwide follow the Anglo-American ‘scoop and run’ model, with the Franco-German ‘stay and play’ approach influencing certain contexts [6], these frameworks face severe limitations in conflict zones. In such settings, Tactical Combat Casualty Care (TCCC) protocols have been developed to protect both emergency responders and victims [7, 8]. However, in Gaza, the deliberate targeting of EMS personnel and infrastructure has rendered even these protective measures insufficient, contributing to the collapse of the emergency medical system [1].

Patients medically evacuated from Gaza to Türkiye may be seen as relatively fortunate compared to civilians who remained in the region. However, factors such as inadequate access to medical histories of patients with chronic conditions, language barriers between evacuated patients and healthcare providers, and profound psychosocial trauma have adversely impacted emergency care [9, 10]. Moreover, for vulnerable groups like the elderly and trauma patients, prolonged emergency department stays have been associated with increased mortality risk [11]. These findings reveal that the hardships faced by war victims continue even after evacuation across borders. While existing literature addresses general barriers to healthcare access in conflict zones, studies focusing specifically on the experiences and access challenges of war-affected individuals medically evacuated from areas under siege, as the case of Gaza Strip, remain scarce.

This study aims to fill a critical gap by examining the experiences of war victims medically evacuated from Gaza to Türkiye in accessing EMS under conflict conditions. Drawing on participants’ testimonies, it identifies physical, structural, and rights-based barriers throughout the process. Specifically, the study investigates the associations between prehospital access times and emergency department treatment durations, examines how evacuation timing and length of stay in Gaza relate to care in Türkiye, and quantifies the prevalence of access-related challenges—including ambulance inaccessibility, prolonged delays, and inadequate infrastructure.

## Methods

### Study design, settings and participants

This descriptive, cross-sectional field study was conducted with Palestinian civilian patients who were medically evacuated from the Gaza Strip to Türkiye for treatment in Ankara following the conflict on October 7, 2023. The study was designed and reported in full compliance with the Strengthening the Reporting of Observational Studies (STROBE) guidelines for observational epidemiological research.

Participants were included on a voluntary basis if they were aged 18 or older, had been medically evacuated from Gaza due to conflict-related conditions, required EMS while in Gaza, and were physically and mentally capable of participating in an interview. Individuals under the age of 18, those who were uncommunicative due to clinical or cognitive conditions, and those who declined participation were excluded. Participant selection was carried out using purposive sampling.

During the study period (November 1, 2024-January 30, 2025), 94 patients were approached and invited to participate, of whom 81 agreed (response rate: 86.1%). The primary reasons for non-participation among the remaining 13 individuals included inability to communicate (*n* = 1), unsuitable clinical condition (*n* = 9), and lack of willingness (*n* = 3).

Given the descriptive nature of the study, a formal sample size calculation was not conducted. Instead, all individuals meeting the inclusion criteria during the study period were enrolled.

### Data collection and implementation

Data were collected using a structured questionnaire developed by the principal investigator in consultation with three domain experts (emergency medicine, public health, and health law) to ensure content validity. While content validity was ensured through expert consultation and pilot testing, formal psychometric validation—such as internal consistency (e.g., Cronbach’s alpha) or test-retest reliability—was not conducted. Existing validated instruments were reviewed during the development process; however, no available scale sufficiently addressed the unique intersection of emergency medical evacuation and healthcare access barriers in conflict zones. The questionnaire consisted of four main sections (Supp. 1):

1. Sociodemographic information.
2. Medical history and comorbidities.
3. Experiences of accessing emergency healthcare in Gaza, intervention processes, and structural barriers.
4. Human rights violations in the context of EMS.

Prior to implementation, the questionnaire was piloted with five patients to assess comprehensibility, timing, and technical adequacy. The final version was administered through face-to-face interviews by trained physicians with experience in humanitarian settings. Interviewers received orientation on trauma-informed and ethically sensitive communication. Questionnaires were available in Turkish, Arabic, and English; participants responded in their preferred language, with support from Arabic-speaking medical interpreters when needed.

### Ethical approval

The study was approved by the Health Sciences Ethics Committee of Ankara Yıldırım Beyazıt University (Ethics Approval No: 07/838; Date: 26.09.2024). Written informed consent was obtained from all participants prior to data collection. No clinical intervention was conducted; data were gathered solely through surveys.

### Statistical analysis

Data were analyzed using Statistical Package for the Social Sciences (SPSS 22.0). Descriptive statistics were reported as counts (n) and percentages (%) for categorical variables, and medians with interquartile ranges (Q1–Q3) for continuous variables. The normality of distribution was assessed using the Kolmogorov-Smirnov and Shapiro-Wilk tests. For group comparisons, the Mann-Whitney U test was used for non-normally distributed data, while the Chi-square test was used for categorical variables. Relationships between variables were examined using Spearman’s correlation analysis. A *p*-value of < 0.05 was considered statistically significant.

The threshold for emergency department (ED) treatment time (< 6 h vs. ≥6 h) was determined based on the median value in the sample. Participants reporting exactly 6 h were categorized under the ≥ 6-hour group for consistency. Comparison groups were clearly defined in each analysis (e.g., income ≤ 500 USD vs. >500 USD). Given the exploratory design and limited sample size, only univariate and bivariate analyses were conducted. The absence of multivariable models (e.g., logistic regression) is acknowledged as a limitation, and future studies are encouraged to adjust for potential confounders such as age, sex, comorbidities, and income level.

## Results

The study included 81 participants (60.5% female) with a median age of 26 years. Most participants reported low monthly income (≤ 500 USD: 80.2%) and had stayed in Gaza for at least one month prior to medical evacuation, with 29.6% reporting stays longer than 120 days. The most frequently reported health conditions were malignancy (35.4%) and extremity trauma (34.1%), while 23.2% had at least one comorbidity, most commonly hypertension (18.6%) or diabetes (11.0%).

When exploring how socioeconomic status relates to access to EMS, participants with a monthly income of ≤ 500 USD experienced significantly longer delays in obtaining care compared to those with higher income levels (*p* = 0.005).

**Table 1:** Sociodemographic and clinical characteristics of participants

Variables	Items	*n*	%
Gender	Female Male	49 32	60.5 39.5
Age	Median (range)	26 (18–46)	
Marital status	Single Married	41 40	50.6 49.4
Education status	Primary Secondary High-school University +	4 11 41 25	4.9 13.6 50.6 30.9
Monthly income (USD)	≤ 500 500–1000 1000–2000 > 2000	65 13 1 2	80.2 16.0 1.2 2.5
Stay in Gaza (months)	Median (Q1-Q3)	3 (2–15)	
Stay in Gaza (days)	0–15 16–30 31–60 61–120 ≥ 120	1 14 38 4 24	1.2 17.3 46.9 4.9 29.6
Stay in Türkiye (months)	1–3 3–6 ≥ 6 Still hospitalized	31 16 13 21	38.3 19.8 16.0 25.9
Health problems*	Median (Q1-Q3)	1 (1–2)	
	Head trauma Chest and abdominal trauma Extremity trauma Malignite Other	13 9 28 29 25	15.9 10.9 34.1 35.4 29.5
Comorbidities*	None Diabetes mellitus (DM) Hypertension (HT) Congestiv Heart Failure	63 9 15 4	76.8 11.0 18.6 4.8
Total		81	100

%: frequency, Q1-Q3: 1st and 3rd quartiles, *: Multiple response options possible

Details regarding access to and referral processes for EMS within Gaza and for international medical evacuation to Türkiye are presented in Table 2. The most significant barrier to accessing emergency care was perceived as the deliberate targeting of healthcare facilities. The most severe health consequence of failing to reach emergency departments was the emergence of life-threatening conditions. The leading cause of patient referral was found to be medication shortages. Language barriers, often due to non-Arabic-speaking personnel during international referral processes, were the most frequently reported challenge.

In terms of emergency medical intervention characteristics, 34.5% (*n* = 28) of patients reported that ambulance services never reached them, and 33.3% (*n* = 27) were never able to access hospital emergency departments. When assessing the infrastructural adequacy of EMS in Gaza, only 25.9% (*n* = 21) of participants considered operating theaters to be sufficient.

The most common reason for premature discharge to continue treatment outside the hospital was physical and medical inadequacy—primarily due to a lack of beds and medications—reported by 49.4% (*n* = 40) of the participants. The most prevalent deficiencies in emergency departments were related to equipment and medical supplies, cited by 87.6% (*n* = 71) of respondents.

**Table 2:** Access to emergency health Services, intervention Processes, and infrastructure adequacy

Variables	Items	*n* *n*	%
Barriers to accessing EMS*	Targeting of healthcare facilities Lack of medical personnel Targeting of ambulances Phones being out of service area	60 51 52 49	74.0 62.9 64.1 60.4
Adequate ambulance equipment availability	Yes No	5 76	6.1 93.8
Most critical health outcomes of not reaching ED*	Life-threatening conditions Permanent disability or damage Chronic diseases	62 34 19	76.5 41.9 23.4
Adequacy of hospital emergency services	Adequate Inadequate	3 78	3.7 96.3
Reasons for international referral*	Inadequate service provision Need for advanced diagnostics Need for surgical treatment Lack of medical equipment Medication shortage Collapse of the healthcare system	64 55 58 65 66 63	79.0 67.9 71.6 80.2 81.4 77.7
Difficulties during international referral*	Financial issues Transportation issues Permit issues Language barrier Overwhelming number of injured Infectious disease Climate-related factors	25 17 15 31 19 12 17	30.8 20.9 18.5 38.2 23.4 14.8 20.9
Ambulance wait time	0–15 min 16–30 min 31–60 min 61–120 min ≥ 120 min Never arrived	13 15 5 12 8 28	15.9 18.3 6.1 14.6 9.8 34.5
Time to reach hospital emergency department (ED)	0–15 min 16–30 min 31–60 min 61–120 min ≥ 120 min Never accessed	11 15 10 13 5 27	13.4 18.3 12.2 15.9 6.1 33.3
Number of deaths due to inability to reach ED	1–5 6–10 More than 10	32 10 39	39.5 12.3 48.1
Are operating rooms adequate for emergency patients?	Yes No	21 60	25.9 74.1
Reasons for early discharge to complete treatment externally	Not discharged Physical and medical inadequacy Forced discharge	25 40 16	30.9 49.4 19.8
Deficiencies in emergency services*	Lack of medical personnel Equipment and supply shortage Medication shortage Lack of empty beds Continuous power outages	60 71 66 58 58	74.1 87.6 81.4 71.6 71.6

%: frequency, *: Multiple response options possible

**Fig. 1:**
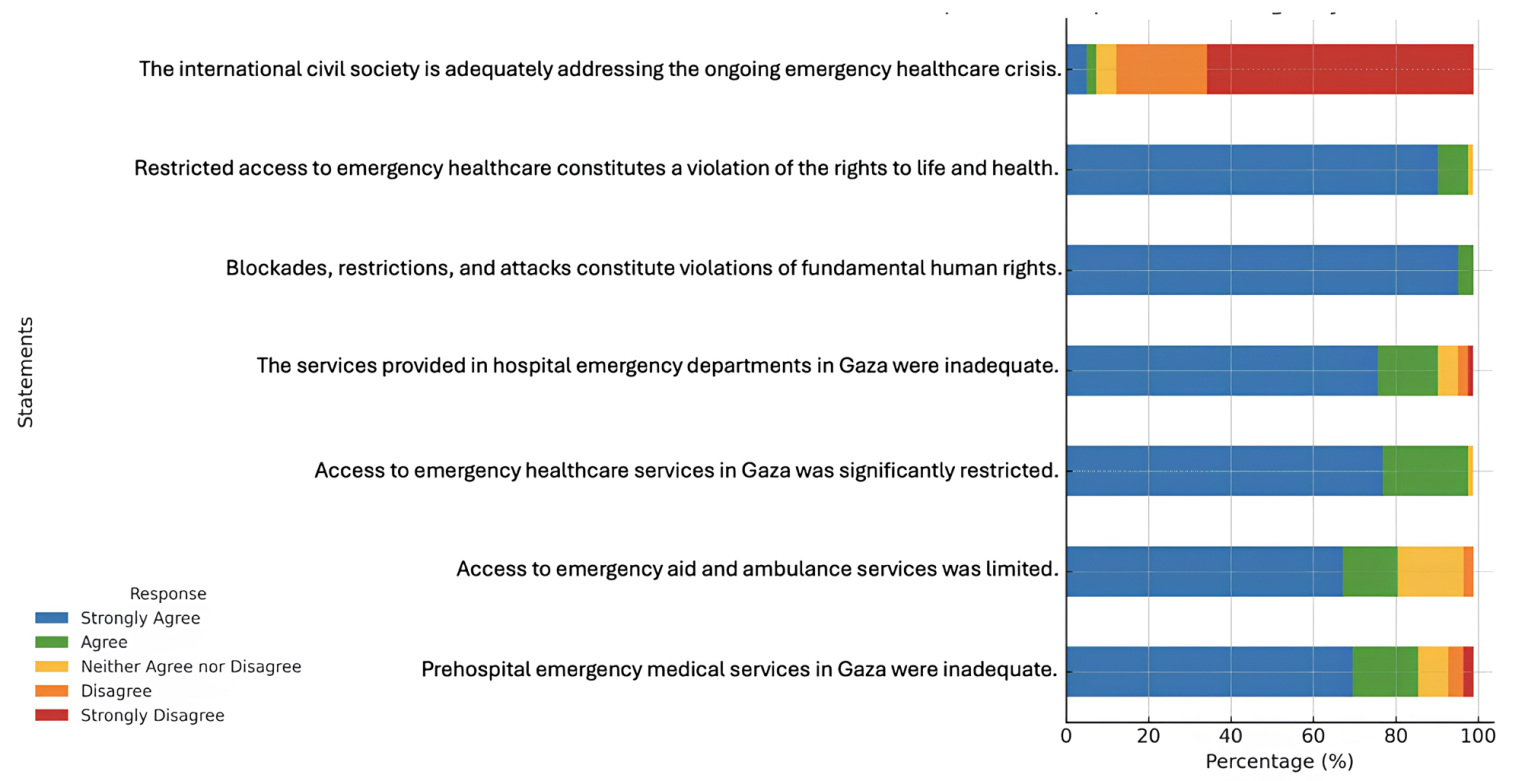
Participants’ Perceptions of Emergency Healtcare Services in Gaza

According to participants’ assessments of healthcare services in Gaza, the most strongly endorsed statement was “Siege, restrictions, and attacks constitute a fundamental violation of human rights,” with 95.1% (*n* = 78) expressing strong agreement. In contrast, the statement “The international civil society is adequately addressing the health crisis” received the highest level of rejection, with 64.6% (*n* = 53) of respondents strongly disagreeing. These findings, illustrated in Fig. 1, reflect a profound sense of injustice regarding the impact of the ongoing siege and military actions on health infrastructure, as well as widespread disillusionment with the global community’s response to the humanitarian crisis in Gaza.

According to the data on human rights violations in EMS, 61.7% (*n* = 50) of participants reported direct exposure to armed or explosive violence, while 37.0% (*n* = 30) experienced attacks by military forces during hospitalization; however, the specific locations or healthcare facilities where these incidents occurred were not recorded. Furthermore, 66.7% (*n* = 54) developed complications due to inadequate medical care. The right to life was the most frequently reported human rights violation, cited by 96.2% (*n* = 78) of respondents.

Despite the widespread occurrence of such violations, only 6.2% (*n* = 5) of patients were able to report them to any organization. When asked about international mechanisms for documenting these violations, 90.1% (*n* = 73) recommended that Israel should be tried in an international court, while 88.8% (*n* = 72) stated that the World Health Organization (WHO) should formally document Israel’s obstruction of healthcare services (Table 3).

**Table 3:** Human rights violations related to access to emergency healthcare

Variables	Items	*n*	%
Direct exposure to armed/bomb attacks	Direct Indirect	50 31	61.7 38.3
Rights violated due to attacks*	Right to life	78	96.2
	Right to health	70	86.4
	Right to movement and residence	73	90.1
	Personal safety and integrity	70	86.4
	Freedom of religion and conscience	63	77.7
Exposure to attacks during hospitalization	Yes No	30 51	37.0 63.0
Complications due to inadequate care	Yes No	54 27	66.7 33.3
Causes of complications	Infection Lack of medication and equipment Amputation	28 16 10	51.8 29.6 18.6
Reported violations to any institution	Yes No	5 76	6.2 93.8
Suggested international responses*	Israel should be prosecuted internationally	73	90.1
	WHO should document Israel’s obstruction	72	88.8

%: frequency, *: Multiple response options possible

Compared to those who waited 30 min or less, patients who waited more than 30 min for an ambulance in Gaza had significantly longer treatment durations in the emergency department (ED) (*p* = 0.036). Patients who reached the ED in more than 30 min had significantly shorter treatment durations compared to those who arrived in 30 min or less (*p* = 0.026). Longer waiting times for ambulance arrival (*p* = 0.013) and ED access (*p* = 0.001) were observed among patients with comorbidities, indicating a potential association without implying causation (Table 4).

**Table 4:** Relationship between emergency healthcare access times and clinical/comorbidity status

		ED treatment		
		**< 6 h** **n (%)**	≥**6 h** **n (%)**	**p**
Ambulance wait time	≤ 30 min > 30 min	18 (54.5) 15 (45.5)	10 (50.0) 10 (50.0)	**0.036**
Time to reach ED	≤ 30 min > 30 min	14 (42.4) 19 (57.6)	12 (57.1) 9 (42.9)	**0.026**
		**Comorbidity**		
		**No** **n (%)**	**Yes** **n (%)**	**p**
Ambulance wait time	≤ 30 min > 30 min	27 (60.0) 18 (40.0)	1 (12.5) 7 (87.5)	**0.013**
Time to reach ED	≤ 30 min > 30 min	26 (59.1) 18 (40.9)	0 (0) 10 (100)	**0.001**

%: frequency, *p*-value determined according to the Chi-square test

## Discussion

This study reveals how conflict functions not only as a source of physical destruction but also as a weapon that systematically paralyzes healthcare services. These documented experiences of civilians in Gaza who were unable to access emergency medical care and were consequently evacuated to Türkiye represent not only a public health crisis but also a direct violation of the rights to life and health. Participants’ reports suggest a perception that healthcare infrastructure was deliberately targeted and that EMS may have been obstructed or withheld as part of the conflict dynamics. Such practices align with the concept of “medicide,” a term used in literature to describe the strategic use of healthcare disruption as a tool of war [12].

The mean age of participants was 26 years (range: 18–46), which is significantly lower than the mean age of 68 reported by El Jabari et al. (2024) [13]. This discrepancy can be explained by the inclusion in this study not only of oncology patients but also trauma victims. A considerable proportion of participants required emergency care for either malignancies or extremity trauma. These findings indicate that under wartime conditions, the demand for emergency healthcare extends beyond acute injuries and includes chronic, complex, and long-term conditions that also require urgent medical attention. Indeed, Mohammed et al. (2024) reported that nearly 2,000 patients in Gaza lost access to treatment following the closure of the territory’s only oncology hospital due to fuel shortages [1].

The WHO defines equal, continuous, and unimpeded access to healthcare as a fundamental human right. However, a study by Bouquet et al. (2021) found that oncology patients in Gaza face not only barriers within their own healthcare system but also substantial obstacles in obtaining the required official permits from Israeli authorities for medical referrals abroad [14]. Among 655 patients included in that study, most initial applications were either denied or delayed, and only 386 were approved. The average delay in reaching the hospital for those approved was 77 days, which had severe adverse effects on disease progression, pain management, treatment continuity, and mental health. Similarly, our findings revealed that patients with comorbid conditions were more likely to report prolonged waiting times for ambulance services and ED access. While this suggests a potential association, the direction of this relationship remains unclear and causality cannot be inferred. Correlation analysis also showed that participants who remained longer in Gaza tended to have longer treatment durations in Türkiye. These findings suggest that access to emergency care in conflict settings may influence treatment outcomes and perceived survival chances.

Socioeconomic inequalities are among the key determinants that shape access to healthcare in conflict settings. In our study, individuals with a monthly income below 500 USD reported significantly more barriers to accessing care compared to those earning more than 500 USD per month, a finding consistent with previous research suggesting that low-income refugee populations may face limited access to healthcare and medication [1, 15]. Furthermore, several participants reported experiencing financial hardship during the referral process. Additionally, individuals with comorbidities experienced significantly longer ambulance wait times and delays in emergency department admissions compared to those without comorbidities. These results suggest that economic disadvantage and medical complexity may jointly contribute to difficulties in accessing emergency care, as perceived by participants.

The most frequently reported access barriers included the deliberate targeting of healthcare facilities, ambulance shortages, and the lack of medical supplies. Participants’ responses reflect not only the perceived fragility of the health system, but also a belief that healthcare infrastructure may have been intentionally undermined as part of the broader conflict. Studies by El Jabari et al. (2024) and Wispelwey et al. (2024) have clearly documented that such practices aim to collapse the Palestinian healthcare system [13, 16]. Consistent with these reports, our findings show that patients in Gaza were unable to receive adequate care due to the collapse of healthcare services, lack of advanced diagnostics and treatment, and shortages of medical equipment and medications, necessitating their evacuation and referral to Türkiye.

A considerable number of patients indicated that they had never received ambulance services, while many reported being unable to reach hospital emergency departments. Notably, nearly half of the participants recounted witnessing multiple deaths they believed were associated with the lack of access to emergency healthcare. These self-reported observations suggest that perceived deficiencies in access may be associated with fatal consequences; however, due to the study’s design and measurement limitations, causal relationships between healthcare access and mortality cannot be established. This aligns with the findings by Nelson et al. (2005) and Venugopal et al. (2007) that underscored the life-threatening consequences of inadequate access to emergency medical care during armed conflict [17, 18].

One of the most commonly reported structural barriers during the international referral process was the language barrier, because many international humanitarian workers did not speak Arabic. This impeded effective communication between healthcare professionals and patients, disrupting diagnostic accuracy, treatment planning, and in-hospital navigation. In a study focused on refugee populations, Cansızlar and Beydağ (2022) also emphasized that language limitations create uncertainty in clinical decision-making, jeopardize patient safety, and directly reduce the quality of healthcare services [19]. Therefore, healthcare teams serving patients evacuated from conflict zones must work with qualified interpreters to ensure clinical effectiveness and uphold ethical standards.

Many participants reported experiencing complications due to insufficient medical care. These complications often involved inadequate treatment in hospitals, the development of infections, and resulting clinical conditions such as amputations that led to permanent disabilities. Similarly, Aldabbour et al. (2024) reported that the shortage of specialized healthcare personnel and essential medical equipment in Gaza significantly hindered the optimal management of trauma and neurological cases [20]. In the present study, deficiencies such as lack of medical staff, shortages of medications and equipment, the unavailability of hospital beds, and frequent power outages were frequently perceived by participants as contributing to poor treatment and recovery experiences.

A considerable number of participants expressed dissatisfaction with the international community’s response to the health crisis in Gaza. This perspective is not limited to individual opinions; Beiraghdar et al. (2023) also highlighted the limited visibility and ineffectiveness of interventions by international organizations such as the WHO and the United Nations (UN) in addressing the multifaceted humanitarian crisis in Gaza [2]. The inaction of international mechanisms in safeguarding health services during wartime creates an environment where violations of civilians’ fundamental health rights remain largely invisible and unpunished.

In this context, a considerable proportion of participants reported violations of the right to life, and the right to health. Notably, a portion stated they were directly attacked within hospital settings, underscoring that the conflict extends beyond the front lines into healthcare facilities themselves. Studies by Levy et al. (2024) and Hamshari et al. (2024) have described the obstruction of access to healthcare in Gaza as a clear breach of international humanitarian law [21, 22]. In our study, a substantial proportion of participants called for Israel to be prosecuted in international courts, while most believed the WHO should be held accountable for documenting Israel’s obstruction of healthcare access. These responses appear to reflect not only individual outcries but also a concrete call for accountability grounded in international humanitarian law. Supporting this, Venugopal et al. emphasized that safeguarding healthcare services during war and disasters is not solely the responsibility of nation-states, but also a principal obligation of international organizations [18].

Although the psychosocial conditions of patients with malignancies should also be assessed, in a context where even basic medical access is largely unattainable, the need for psychological support has become secondary. In conclusion, this study comprehensively illustrates the inequalities in healthcare access during wartime and the destructive impact of conflict on healthcare systems. When interpreted alongside with literature, our results highlight that protecting EMS and implementing specialized health policies for war victims are not only ethical and humanitarian imperatives but also legal obligations [1, 15]. Although this study primarily focused on physical health access barriers, the profound psychological distress experienced by patients—including trauma, anxiety, and uncertainty—warrants further investigation in future research.

These findings offer several actionable insights for improving access to emergency medical care in conflict zones. First, the deployment of mobile field hospitals may address local infrastructure collapse. Second, multilingual healthcare teams or trained interpreters should be included in international missions to overcome language barriers. Third, international organizations must enhance coordination for medical evacuations and ensure neutral monitoring of access violations. Lastly, legal accountability mechanisms must be established to document and respond to systematic obstruction of healthcare services. These measures can help bridge the gap between humanitarian principles and real-world practice in war-affected regions.

### Limitations

While this study provides critical insights into access to EMS in a conflict setting, it is subject to several methodological limitations. First, the research exclusively includes patients who were medically evacuated to Türkiye and treated at a specific healthcare institution. Consequently, the experiences of individuals who remained in Gaza or received treatment in other countries are not represented, which limits the generalizability of the findings. These individuals likely represent a specific subset of patients who were eligible and prioritized for international evacuation, which may not reflect the broader population affected by the conflict.

Second, since the data rely on participants’ self-reports, the objectivity of responses may be partially influenced by post-traumatic cognitive effects or potential recall bias. Third, the use of interpreter assistance in some interviews may have led to semantic distortions or nuanced discrepancies in language translation. Moreover, although the study includes analytic comparisons, its cross-sectional design, measurement limitations, and non-representative sample preclude any causal interpretations. The findings should therefore be considered exploratory and hypothesis-generating, offering a descriptive snapshot of conditions during a specific period. Additionally, the study lacks baseline or pre-conflict data, limiting the ability to assess how access barriers evolved over time or differed from pre-war healthcare conditions.

Furthermore, although the questionnaire was developed with expert consultation and piloted for clarity, no formal psychometric validation (e.g., internal consistency or reliability testing) was conducted, which may affect measurement precision.

Lastly, the analysis was limited to univariate and bivariate comparisons due to sample size constraints. The lack of multivariable modeling restricts the ability to adjust for potential confounders such as age, sex, comorbidities, or socioeconomic status.

## Conclusions

This study highlights participants’ reports suggesting that the ongoing conflict in Gaza may have deliberately impacted the healthcare system, rendering EMS inaccessible and severely undermining civilians’ rights to life and health. The self-reported experiences of participants who were unable to access ambulances or emergency departments and who received inadequate care suggest a perceived intentional incapacitation of healthcare services. These accounts may reflect a systematic pattern of violations that, from the participants’ perspective, aligns with the concept of “medicide” as described in the literature.

The findings emphasize the importance of protecting EMS neutrality in conflict settings as a fundamental medical and legal obligation. Participants’ testimonies highlight that failures in EMS provision are not seen merely as local service gaps but as indicators of broader ethical and humanitarian breakdowns. In this light, there is a perceived need—expressed by participants—for international organizations, particularly the WHO and UN, to establish and enforce mechanisms that protect EMS from interference during armed conflict.

Furthermore, this study highlights specific barriers reported by participants, including ambulance shortages, prolonged delays, denial of ED access, and infrastructure damage. These insights may inform targeted strategies such as mobile field hospital deployment, improved medical evacuation protocols, and multilingual support systems. While the study cannot empirically establish causality or generalizability, its findings offer valuable direction for future humanitarian planning and policy development.

## Supplementary Information

Below is the link to the electronic supplementary material.

Supplementary Material 1

## Data Availability

The datasets used and analyzed during the current study are available from the corresponding author upon reasonable request.

## Abbreviations

ED: Emergency department
TCCC: Tactical combat casualty care
UN: United nations
WHO: World health organization
