# Supplementary material for: Access to emergency medical services and associated barriers among war-affected patients evacuated from Gaza: a cross-sectional study
Source: Arch Public Health. 2025 Oct 1;83:234. doi: 10.1186/s13690-025-01734-w (PMC12490137; doi:10.1186/s13690-025-01734-w)
Supplement: Supplementary file 1 — Supplementary Material 1 [file 13690_2025_1734_MOESM1_ESM.docx]

**Supplementary Material 1. - Survey Questionnaire**

**SECTION A: Sociodemographic Characteristics**

1. **Gender:**
   ☐ Female  ☐ Male
2. **Age:** ___ years
3. **Marital Status:**
   ☐ Single  ☐ Married  ☐ Divorced  ☐ Widowed
4. **Educational Attainment:**
   ☐ Primary ☐ Secondary ☐ High school ☐ University or above
5. **Occupation:** _______________________________________
6. **Monthly Household Income (in USD):**
   ☐ ≤500  ☐ 501–1000  ☐ 1001–2000  ☐ >2000

**SECTION B: Clinical and Evacuation Information**

1. **Primary Health Condition(s) for Treatment (Select all that apply):**
   ☐ Head trauma
   ☐ Chest or abdominal trauma
   ☐ Extremity trauma
   ☐ Malignancy (e.g., cancer)
   ☐ Other: ____________
2. **Comorbidities (Select all that apply):**
   ☐ None
   ☐ Diabetes Mellitus
   ☐ Hypertension
   ☐ Congestive Heart Failure
   ☐ Other: ____________
3. **How long did you stay in Gaza after October 7, 2023?**
   ☐ 0–15 days  ☐ 16–30 days  ☐ 31–60 days
   ☐ 61–120 days  ☐ More than 120 days
4. **How long did it take you to reach Türkiye for treatment after evacuation began?**
   ☐ 1 day ☐ 3 days ☐ 1 week ☐ 2 weeks or more
5. **How long have you been in Türkiye for medical treatment?**
   ☐ 1–3 months ☐ 3–6 months ☐ More than 6 months ☐ Still hospitalized

**SECTION C: Access to Emergency Medical Services in Gaza**

1. **Did ambulance services reach you during your emergency?**
   ☐ Yes ☐ No
2. **If yes, how long did you wait for an ambulance to arrive?**
   ☐ 0–15 minutes ☐ 16–30 minutes ☐ 31–60 minutes
   ☐ 61–120 minutes ☐ More than 120 minutes ☐ Never arrived
3. **Were you able to access a hospital emergency department?**
   ☐ Yes ☐ No
4. **If yes, how long did it take to reach the ED?**
   ☐ 0–15 minutes ☐ 16–30 minutes ☐ 31–60 minutes
   ☐ 61–120 minutes ☐ More than 120 minutes ☐ Never accessed
5. **How long did you receive treatment in the ED?**
   ☐ Less than 6 hours ☐ 6 hours or more
6. **What barriers did you face in accessing emergency services? (Select all that apply)**
   ☐ Ambulances targeted
   ☐ Health facilities targeted
   ☐ Shortage of medical personnel
   ☐ Phones out of service area
   ☐ Other: ____________
7. **What were the consequences of failing to reach the ED? (Select all that apply)**
   ☐ Life-threatening condition
   ☐ Permanent disability
   ☐ Chronic condition
   ☐ No serious consequence

**SECTION D: Infrastructure and Referrals**

1. **Are ambulance services adequately equipped?**
   ☐ Yes ☐ No
2. **Are hospital operating rooms adequate for emergency care?**
   ☐ Yes ☐ No
3. **Were you discharged early to continue treatment elsewhere?**
   ☐ No
   ☐ Yes, due to lack of beds or medications
   ☐ Yes, by force (e.g., military discharge)
4. **What deficiencies did you notice in hospital emergency services? (Select all that apply)**
   ☐ Lack of personnel
   ☐ Equipment and supply shortages
   ☐ Medication shortage
   ☐ Lack of beds
   ☐ Power outages ☐ Other: ____________
5. **Reasons for international referral (Select all that apply):**
   ☐ Inadequate services
   ☐ Need for advanced diagnostics
   ☐ Need for surgery
   ☐ Medication shortage
   ☐ Collapse of health system
   ☐ Other: ____________
6. **Difficulties faced during international referral (Select all that apply):**
   ☐ Financial
   ☐ Transportation
   ☐ Permit issues
   ☐ Language barrier
   ☐ Infectious disease
   ☐ Overcrowding
   ☐ Climate/environmental reasons ☐ Other: ____________

**SECTION E: Human Rights & Perceptions**

1. **Were you directly exposed to bombings or attacks?**
   ☐ Yes ☐ No
2. **Were you attacked during hospitalization?**
   ☐ Yes ☐ No
3. **Do you believe any of the following rights were violated? (Select all that apply)**
   ☐ Right to life
   ☐ Right to health
   ☐ Right to movement and residence
   ☐ Personal safety and integrity
   ☐ Freedom of religion and conscience
   ☐ None
4. **Did you experience any complications due to inadequate care?**
   ☐ Yes ☐ No
5. **If yes, what were the complications? (Select all that apply)**
   ☐ Infection
   ☐ Lack of medication/equipment
   ☐ Amputation
   ☐ Other: ____________
6. **Did you report these violations to any authority or organization?**
   ☐ Yes ☐ No
7. **What should be the international response? (Select all that apply)**
   ☐ WHO should document these violations
   ☐ Israel should be tried in international court
   ☐ Other: ____________

**SECTION F: Perceptions**

**32. Please indicate your level of agreement with the following statements:**

| **Statement** | **Strongly Disagree** | **Disagree** | **Neither Agree nor Disagree** | **Agree** | **Strongly Agree** |
| --- | --- | --- | --- | --- | --- |
| The siege and attacks are a fundamental human rights violation. | ☐ | ☐ | ☐ | ☐ | ☐ |
| Prehospital emergency medical services in Gaza were inadequate. | ☐ | ☐ | ☐ | ☐ | ☐ |
| International civil society is adequately responding to the health crisis in Gaza. | ☐ | ☐ | ☐ | ☐ | ☐ |
| Access to emergency healthcare services in Gaza was significantly restricted. | ☐ | ☐ | ☐ | ☐ | ☐ |
| Restricted access to emergency healthcare constitutes a violation of the rights to life and health. | ☐ | ☐ | ☐ | ☐ | ☐ |
| The services provided in hospital emergency departments in Gaza were inadequate. | ☐ | ☐ | ☐ | ☐ | ☐ |
| Access to emergency aid and ambulance services was limited. | ☐ | ☐ | ☐ | ☐ | ☐ |

**SECTION G: Additional Feedback**

1. **Do you have any suggestions or comments regarding emergency medical services or health access in conflict settings?**
